# Cellular adhesome screen identifies critical modulators of focal adhesion dynamics, cellular traction forces and cell migration behaviour

**DOI:** 10.1038/srep31707

**Published:** 2016-08-17

**Authors:** Michiel Fokkelman, Hayri E. Balcıoğlu, Janna E. Klip, Kuan Yan, Fons J. Verbeek, Erik H. J. Danen, Bob van de Water

**Affiliations:** 1 Leiden Academic Centre for Drug Research, Division of Toxicology, Leiden University, The Netherlands; 2 Imaging & Bioinformatics, Leiden Institute of Advanced Computer Science, Leiden University, The Netherlands

## Abstract

Cancer cells migrate from the primary tumour into surrounding tissue in order to form metastasis. Cell migration is a highly complex process, which requires continuous remodelling and re-organization of the cytoskeleton and cell-matrix adhesions. Here, we aimed to identify genes controlling aspects of tumour cell migration, including the dynamic organization of cell-matrix adhesions and cellular traction forces. In a siRNA screen targeting most cell adhesion-related genes we identified 200+ genes that regulate size and/or dynamics of cell-matrix adhesions in MCF7 breast cancer cells. In a subsequent secondary screen, the 64 most effective genes were evaluated for growth factor-induced cell migration and validated by tertiary RNAi pool deconvolution experiments. Four validated hits showed significantly enlarged adhesions accompanied by reduced cell migration upon siRNA-mediated knockdown. Furthermore, loss of PPP1R12B, HIPK3 or RAC2 caused cells to exert higher traction forces, as determined by traction force microscopy with elastomeric micropillar post arrays, and led to considerably reduced force turnover. Altogether, we identified genes that co-regulate cell-matrix adhesion dynamics and traction force turnover, thereby modulating overall motility behaviour.

Cell migration plays an important role in many physiological processes, such as embryonic development, skin renewal and immune response^1^. Deregulation of this cellular process plays a role in various pathologies, including cancer^2^. Tumour metastasis is the most lethal aspect of cancer progression and involves tumour cell invasion and dissemination^3^,^4^. Moreover, modelling has shown that short-range migration contributes to mixing of cell clones inside the tumour, thereby promoting tumour growth^5^. Thus, oncogenic signalling pathways causing enhanced tumour cell migration *in vitro* may contain candidate targets for blocking tumour growth and metastasis formation *in vivo*.

Cell migration on 2D environments typically consists of several steps: protrusion, attachment, cell body movement and tail retraction^6^,^7^. Cell matrix adhesion dynamics and remodelling of the actin cytoskeleton plays a role in all of these processes^8^,^9^,^10^. Cell protrusions are driven by actin polymerization^11^ and stabilized by attachment of the leading edge to the underlying surface through integrin mediated cell-matrix adhesions. These adhesions contain a dynamic integrin-associated multiprotein complex that locally couples the extracellular matrix (ECM) to the actin cytoskeleton and, through cytoskeletal connections with the nuclear membrane, to the nucleus^10^,^12^. Cell body movement is driven through contractile actomyosin bundles that pull the cell body and nucleus towards the leading edge^13^. Finally the trailing edge is retracted by inducing cell-matrix adhesion disassembly, possibly through microtubule signalling^14^.

Formation of cell-matrix adhesions and the actomyosin contractile machinery have also been shown to mediate some forms of cell migration in 3D^15^. However, the paradigm of 2D cell migration does not translate well to all 3D environments and (tumour) cells show a high level of plasticity allowing them to switch between different modes of migration in 3D^16^,^17^.

Here, we aimed to understand the underlying machinery of tumour cell migration and its relation to adhesion turnover and cellular traction forces. For this purpose we performed a siRNA screen targeting cell adhesion and actin dynamics associated genes, and evaluated their role in cell-matrix adhesion size and/or dynamics. Subsequently we tested the relationship with tumour cell migration as well as cellular traction forces for the most relevant candidate genes. As such, we identified myosin phosphatase target 2 (PPP1R12B), Homeodomain Interacting Protein Kinase 3 (HIPK3) and Ras-related C3 botulinum toxin substrate 2 (RAC2) to be involved in the organization and dynamics of traction forces, the cellular adhesion machinery, and cell motility induced by growth factor signalling.

## Results

### Imaging-based RNAi screening of focal adhesion dynamics

**Figure 1:**
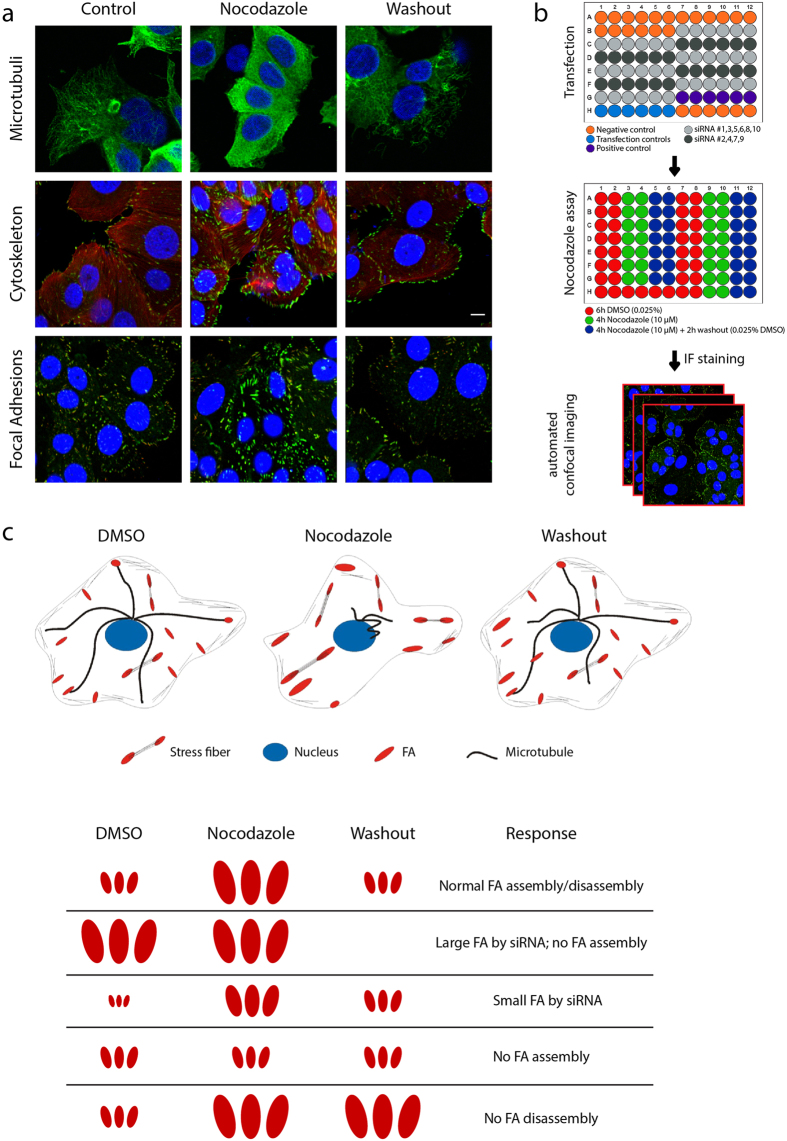
A nocodazole-based microtubule depletion assay to study focal adhesion dynamics in high-throughput fashion. (**a**) Treatment of MCF7 WT cells with nocodazole (10 μM, 4 h) depolymerized microtubules (MT), leading to activation of the actin cytoskeleton and focal adhesion assembly. Washout of nocodazole allows MT regrowth, resulting in focal adhesion disassembly. MCF7 WT cells were stained for vinculin and phosphorylated paxillin to visualize focal adhesions. (**b**) Schematic representation of screen setup. Transfection was performed in 96-well plates and controls were included in each plate. MCF7 WT cells were exposed as indicated, to induce FA assembly and disassembly. Plates were stained for vinculin and phosphorylated paxillin and imaged with automated confocal microscopy. (**c**) Schematic representation of cellular and FA organization in the nocodazole assay, with possible FA phenotypes caused by siRNA knockdown.

We developed a RNAi screen to identify genes that modulate focal adhesion dynamics and thereby tumour cell migration. Focal adhesions (FA) are highly dynamic multi-molecular complexes and despite the advances in time resolution of microscopy systems, live imaging of these highly dynamic structures is not possible in a high-throughput fashion. In order to identify genes that regulate the complex dynamics of focal adhesions, we established a nocodazole-based assay to specifically separate adhesion assembly and disassembly. Purposely we used MCF7 cells that are epithelial in nature and have low motility, allowing the analysis of nocodazole-induced focal adhesion turnover in rather static cells. When cells are exposed to nocodazole, the microtubules depolymerize and release GEF-H1, which in turn activates RhoA, resulting in cell contractility and focal adhesion assembly^18^. Upon nocodazole washout, microtubules regrow towards the cell periphery and induce focal adhesion disassembly^19^. Indeed, after nocodazole treatment, MCF7 cells showed depolymerized microtubules, stress fibre formation and enlarged focal adhesions (Fig. 1a). When nocodazole was washed out, microtubule regrowth induced focal adhesion disassembly, indicating that focal adhesion assembly in this system was reversible (Fig. 1a).

Next, the siRNA transfection, nocodazole assay and automated confocal microscopy were optimized for 96-well plates and high-throughput screening. Each plate contained a set of controls (negative, positive and transfection controls) and 10 different siRNAs (Fig. 1b). Negative controls included no siRNA, mock, non-targeting si-Control #2 and si-GFP. Knockdown of Dynamin 2 was shown to increase focal adhesion size in steady-state conditions and to inhibit microtubule-induced focal adhesion disassembly^19^,^20^, therefore si-DNM2 was used as positive control (Supplementary Fig. S1). As transfection controls si-KIF11, si-PXN and siGLO-green were used, which result in reduced proliferation, reduced intensity of paxillin staining and localized green fluorescent signal in the nucleus, respectively, and allowed visual inspection of transfection efficiency. Knockdown efficiency was determined by Western Blot analysis to confirm successful knockdown (Supplementary Fig. S1). To test for possible edge effects, cells were transfected with control siRNAs and FA size distributions from different wells were compared (Supplementary Fig. S2). No differences were found between wells at the edge or centre of the plate, indicating little well-to-well variation and no edge effect. Transfected cells were subjected to the nocodazole assay (treatment as indicated in Fig. 1b) to study changes in focal adhesion morphology after siRNA knockdown under steady-state conditions (DMSO-condition), and during focal adhesion assembly (nocodazole-condition) and disassembly (washout-condition). After the nocodazole assay, cells were fixed and stained for focal adhesions (phosphorylated Tyr118-paxillin and vinculin) and nuclei. A custom-made macro was used for automated confocal microscopy imaging, which first searched for a minimum of Hoechst positive nuclei per image, then identified the optimal focus followed by image acquisition of focal adhesions. A schematic overview of the anticipated results is illustrated in Fig. 1c. The nocodazole assay allowed us to identify siRNAs that alter focal adhesion morphology under steady-state conditions, as well as siRNAs that specifically disrupt focal adhesion assembly or disassembly.

**Figure 2:**
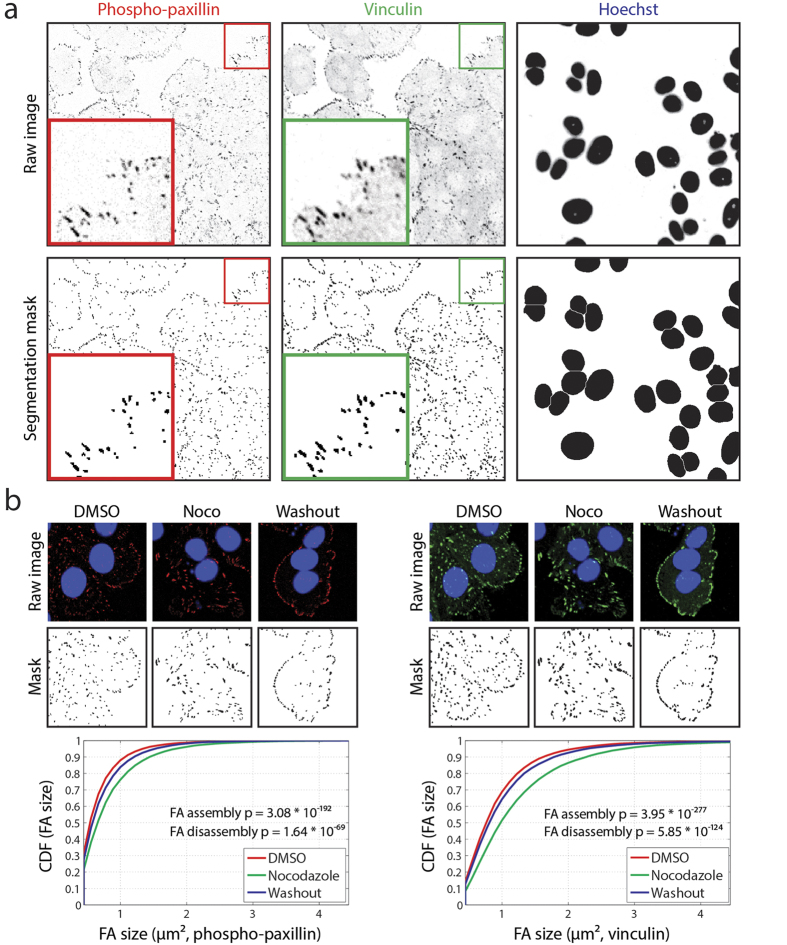
Automated image analysis of focal adhesion morphology allows quantitative assessment of different dynamic states of FAs. (**a**) Automated image analysis identifies individual focal adhesions based on pTyr118-paxillin or vinculin staining. (**b**) Image analysis and quantification of focal adhesion sizes in a set of representative images of the nocodazole assay. Focal adhesion size distribution of nocodazole-induced FA assembly and microtubule-induced FA disassembly were compared using a Kolmogorov-Smirnov (KS) test.

To determine whether siRNA knockdown affected focal adhesion morphology in MCF7 cells, we developed a robust image analysis pipeline in ImageJ to quantify each individual focal adhesion. The analysis was performed on phosphorylated paxillin (pTyr118) staining and vinculin staining, providing quantitative data for both (Fig. 2a). First, the nuclei were segmented, counted, and images containing less than 3 cells were excluded from further analysis. Next, the two focal adhesion stainings were analysed (see Material & Methods for details). Focal adhesions smaller than 4 pixels (0.46 μm^2^) were ignored, ensuring that only bona fide focal adhesions were included in the analysis. An example of the final segmentation mask for pTyr118-paxillin and vinculin is shown in Fig. 2a. To verify the effect of nocodazole-induced focal adhesion assembly, the distribution of focal adhesion size in control cells (DMSO condition) was compared to the focal adhesion size of nocodazole-treated cells (Fig. 2b). Quantitative analysis confirmed that 4 hr nocodazole treatment induced focal adhesion assembly, as shown by the shift in distribution, and that this increase in adhesion size was highly significant (p-value 3.08 * 10^−192^ and 3.95 * 10^−277^, for pTyr118-paxillin and vinculin, respectively). Similarly, focal adhesion size was significantly smaller in the washout condition compared to nocodazole condition (p-value 1.64 * 10^−69^ and 5.85 * 10^−124^, for phosphorylated paxillin and vinculin, respectively), indicating that a 2 hr washout was sufficient to detect focal adhesion disassembly. Furthermore, these results showed that both focal adhesion assembly and disassembly can be induced and subsequently evaluated by quantitative image analysis.

### Primary screen for siRNAs affecting focal adhesion dynamics

**Figure 3:**
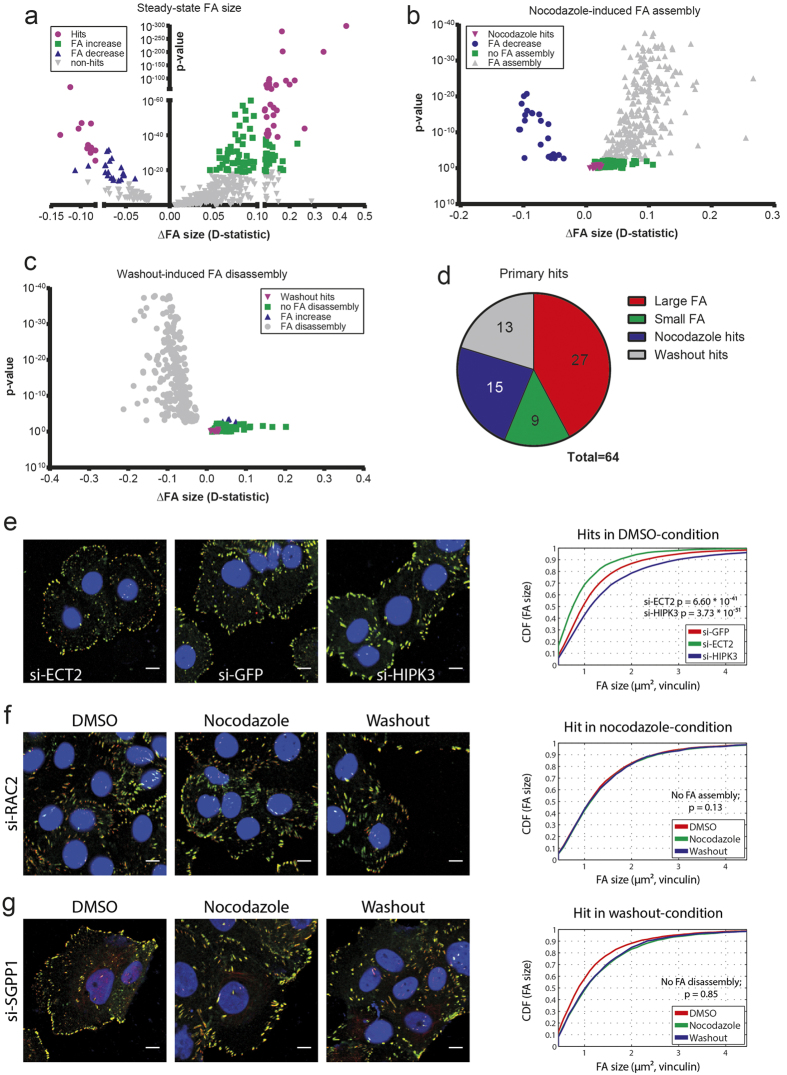
RNAi screen identifies novel regulators of focal adhesion dynamics. (**a**) Focal adhesion size distributions after siRNA knockdown in DMSO condition were compared to siGFP control cells using a two-tailed KS-test. The shift in size distribution (D-statistic) is used as a measurement of change in focal adhesion size. A decrease in focal adhesion size is shown in blue and an increase in size in green. Hits remaining after stringent thresholding are shown in purple. (**b**) Adhesion size of siRNA knockdown cells in nocodazole condition was compared to DMSO of the same siRNA, to detect impaired FA assembly. siRNAs that show no change in FA size were considered as hits (purple). (**c**) Adhesion size of siRNA knockdown cells in washout condition was compared to nocodazole of the same siRNA, to detect impaired FA disassembly. siRNAs that show no change in FA size were considered as hits (purple). (**d**) In total 64 hits were found to affect adhesion morphology under steady state conditions (red and green), or to specifically impair FA assembly (blue) or disassembly (grey). (**e**) Example images of adhesion size decrease (si-ECT2) and increase (si-HIPK3) after siRNA knockdown in DMSO condition. Quantification of FA size is shown on the right. (**f**) Loss of RAC2 inhibited adhesion assembly. Quantification of FA size shows identical distributions in the different conditions. (**g**) Knockdown of SGPP1 impaired adhesion disassembly. Quantification of FA sizes confirms no change in washout condition compared to nocodazole.

To identify genes that regulate focal adhesion dynamics, we performed a high-throughput, imaging-based RNAi screen using a custom designed siRNA library targeting 569 adhesion and cytoskeletal related genes (Supplementary Table 1). Focal adhesion size distributions after siRNA knockdown were compared with siGFP transfected cells, to assess perturbation of adhesion organization under control conditions (Fig. 3a, Supplementary Table 2). A Kolmogorov-Smirnov test (KS-test) was used to compare size distributions, which provided a KS-test score, indicating a significant decrease (−1), increase (+1), or no change (0) in adhesion size, and a p-value. Furthermore, the maximal distance (D-statistic) between distributions was calculated as a measurement of change in adhesion size. This D-statistic and the p-value were used to set thresholds for hit calling and separate thresholds were set for focal adhesion size increase and decrease. A large number of siRNAs showed significant changes in adhesion size, therefore we selected hits that showed the largest effect and were most significant. For focal adhesion size increase, siRNAs required a D-statistic >0.1 and a p-value < 10^−40^ to be considered as hits. For focal adhesion size decrease, hits were defined as siRNAs with a D-statistic <−0.075 and p-value < 10^−25^. An example of each case is given in Fig. 3e. With these thresholds, 30 hits were found to increase focal adhesion size and 11 hits showed reduced adhesion size. CDH11, PXN, ACTG1, TUBA1 and HSPA2 were deselected for further validation experiments because of previous extensive studies in cell motility and/or high overall protein abundance and consequently reduced likelihood as drug target.

To discover genes that specifically affect focal adhesion assembly, the adhesion size of nocodazole-treated knockdown cells was compared to their own DMSO-condition. In siGFP-transfected control cells, induction of focal adhesion assembly was observed following exposure to nocodazole and cells showed enlarged adhesions. Potential hits were defined as siRNAs that inhibited this nocodazole-induced adhesion assembly and showed no adhesion size increase. We selected 15 hits that showed no significant change in focal adhesion size upon nocodazole treatment (Fig. 3b), e.g. loss of RAC2 inhibited adhesion assembly and subsequent disassembly, suggesting a functional role in the dynamic organization of focal adhesions (Fig. 3f).

In this fixed screen setup, focal adhesion disassembly under washout condition is dependent on nocodazole-induced assembly. Without enlargement of adhesions in nocodazole condition, the subsequent disassembly upon washout could naturally not be studied. Therefore, we focused on siRNAs that showed ‘normal’ adhesion assembly in the nocodazole condition, and subsequently defined siRNAs that inhibited washout-induced focal adhesion disassembly as hits (Fig. 3c). Knockdown of SGPP1 showed minimal changes in adhesion size in DMSO condition and showed typical induction of assembly, however adhesion disassembly was severely impaired (Fig. 3g). Ultimately, 13 hits were selected that showed minimal changes in adhesion size compared to their own nocodazole condition. In total, 64 hits were selected after applying stringent thresholds (Fig. 3d, Supplementary Table 3).

### Disrupted focal adhesion organization impairs cell migration

**Figure 4:**
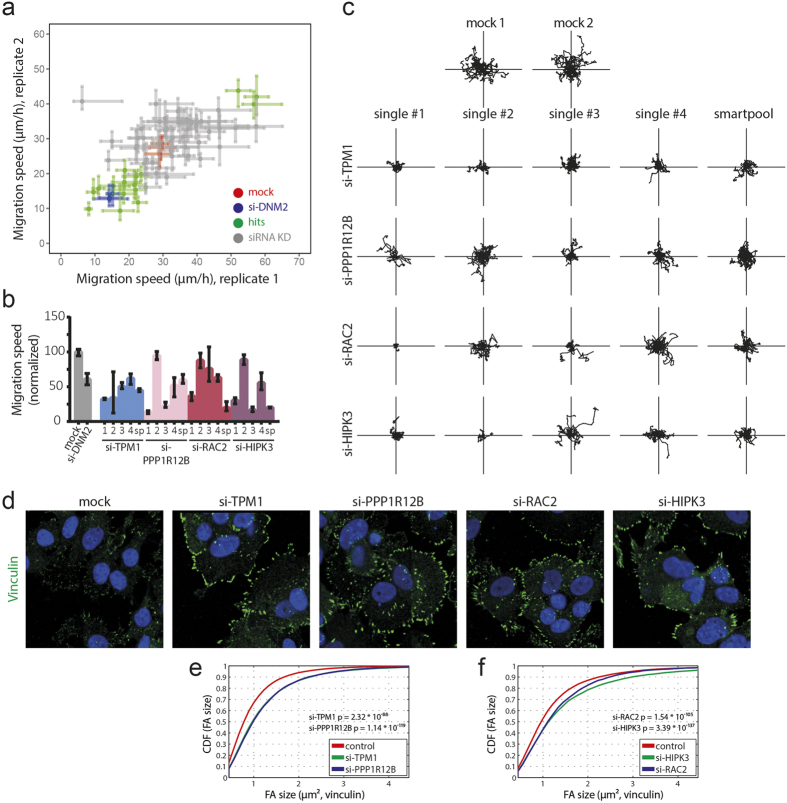
Disrupted FA organization affects cell migration. (**a**) Quantification of single cell migration speed of MCF7-IGF1R cells after knockdown of 64 hits. MCF7-IGF1R cells were transfected with siRNAs and cell migration was assessed by live microscopy. Hits showing significant effects and consistent results in both replicates were considered for validation by single siRNA sequences. Median ± 95% confidence interval is shown and cell populations were compared by Kruskal-Wallis test with Dunn’s post correction test. (**b**) Four single siRNA sequences per gene were evaluated in random cell migration assays and show similar results as smartpool siRNA, thereby validating these hits. Median ± 95% confidence interval was normalized to mock control. (**c**) Single cell trajectories were plotted to confirm inhibition of cell migration by knockdown of different genes with single siRNA sequences. (**d**) Four validated hits showed consistently large focal adhesions in MCF7-IGF1R cells from cell migration experiments. Representative images of 1 of 4 replicates are shown. (**e,f**) Quantification of FA size of cells shown in **d**. All four validated hits show significant induction of FA size, according to KS-test. P-values are shown.

We next defined adhesion-related genes that affect focal adhesion dynamics and consequently also migratory behaviour. To validate our hits and to directly define the relationship between impaired adhesion organization and cell migration, all 64 primary hits were evaluated in a cell migration assay. As MCF7 cells have very limited motility, likely due to strong E-cadherin-mediated cell junction formation, we used MCF7 cells with an ectopic overexpression of the Insulin-like Growth Factor 1 Receptor (MCF7-IGF1R)^21^. These cells show a clear induction of cell migration upon stimulation with Insulin-like Growth Factor 1 (IGF1), which is related to loss of E-cadherin junction and induction of a scattered highly motile cellular phenotype. Importantly, these cells had the same genetic background as the MCF7 cells used in our primary screen and showed similar induction of FA assembly and disassembly in response to nocodazole treatment. MCF7-IGF1R cells were transfected with 64 individual smartpool siRNA mixes and IGF1-stimulated cell migration was visualized by combined live differential interference contrast (DIC) and epi-fluorescence microscopy. Migration of individual MCF7-IGF1R cells was quantified by tracking the nuclei and calculating single cell speeds (Supplementary Fig. S3). Positive control si-DNM2 significantly reduced cell migration when compared to mock condition as expected. Knockdown of 16 candidate genes consistently showed significant modulation of IGF1-induced migration in the replicate experiments (Fig. 4a), with 13 hits demonstrating inhibition of cell migration. This indicates that most of the genes that we identified in our screen and modulate focal adhesion dynamics impair rather than stimulate cell migration. Next we assessed the effect of individual siRNAs that were part of the smartpool for 16 candidate genes. Only 1 hit (ITGB3BP) was confirmed to induce cell migration upon knock down, whereas 10 hits were consistently inhibiting cell migration with 2 single sequences or more (Fig. 4b,c and Supplementary Fig. S4). These hits included: ACTR3, AHRGAP24, CAPN6, CD47, CFL1, HIPK3, KEAP1, PPP1R12B, RAC2 and TPM1. MCF7-IGF1R cells were subsequently stained for adhesion protein vinculin and focal adhesions were imaged and analysed as before. Four candidate genes caught our interest (TPM1, PPP1R12B, RAC2 and HIPK3), showing enlarged adhesions in all experiments (Fig. 4d) and consistent inhibition of IGF1-induced migration of MCF7-IGF1R cells (Fig. 4b,c, Supplemental movies 1,2,3,4,5). Quantification of focal adhesion sizes confirmed a similar change in size as found in the MCF7 WT cells under DMSO conditions (Fig. 4e,f). We further focused on these candidates because of their consistent strong effects, and since for two of them little is known about their involvement in focal adhesion dynamics and cell migration.

### Knockdown of PPP1R12B, RAC2 and HIPK3 results in higher traction forces and slower force turnover

**Figure 5:**
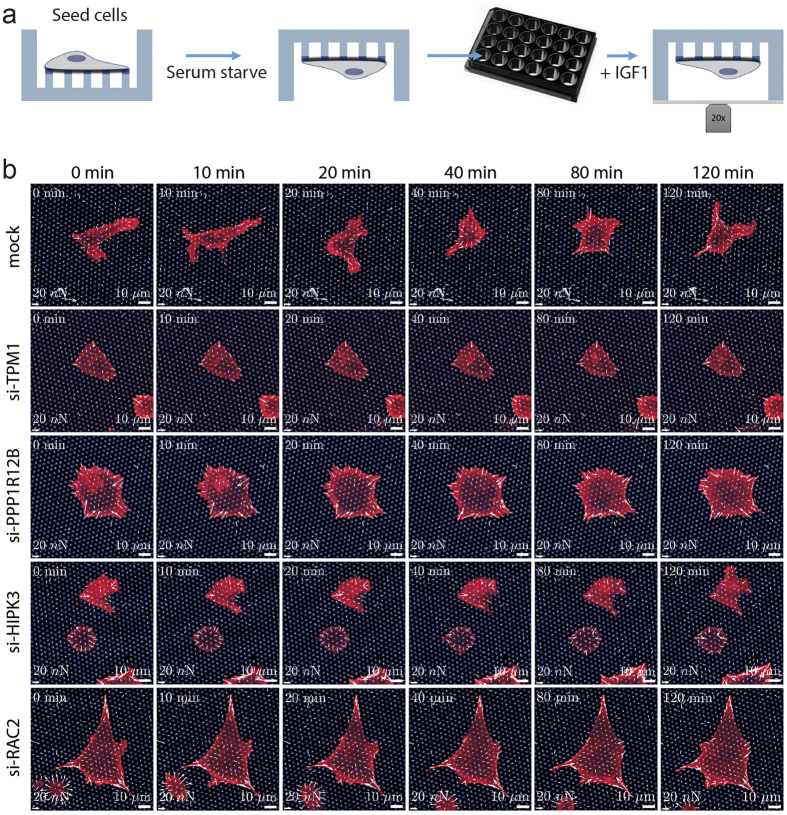
Traction force microscopy reveals a reduced dynamic in knockdown cells. (**a**) Schematic overview of experimental setup. Transfected cells were seeded on top of the micropillars and allowed to adhere, after which cells were briefly deprived of serum. Micropillar arrays were placed upside down in a glass bottom plate and cellular force application was visualized by live confocal microscopy. (**b**) Representative images of temporal force application of RNAi-mediated knockdown cells. White arrows indicate magnitude and direction of forces measured. Scale bar: 20 nN and 10 μm.

**Figure 6:**
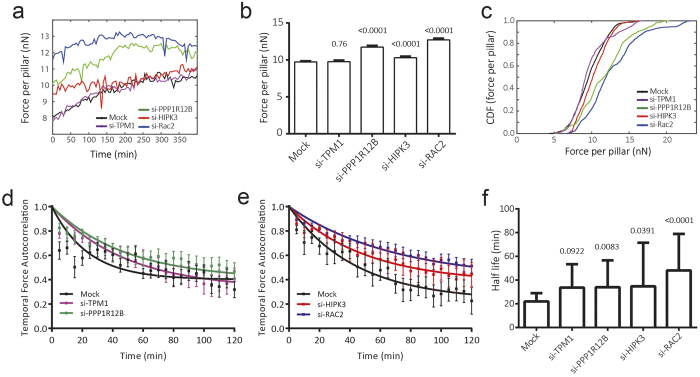
Induced cellular force application and reduced force turnover upon knockdown of PPP1R12B, HIPK3 and RAC2. (**a**) Average force per pillar was calculated for each time point and shows constant force application in each condition. (**b**) Knockdown of PPP1R12B, HIPK3 and RAC2 resulted in significantly higher cellular traction forces, as determined by unpaired t-test with Welch’s correction. Mean ± 95% confidence interval is shown. (**c**) Distribution of measured cell traction forces. (**d,e**) Temporal dynamics of force application is plotted as mean ± SEM autocorrelation coefficient of magnitude of forces on individual pillars over time. A non-linear fit of the relationship indicates that autocorrelation of temporal forces is lower in mock transfected cells, compared to knockdown conditions. (**f**) Halftimes of fitted autocorrelation functions show fast force dynamics in mock-transfected cells, whereas force turnover in knockdown conditions is reduced by approximately 50%. The extra sum of squares F-test was used to compare groups. (**a–f**) Four independent experiments were performed, with each knockdown tested in two independent experiments. The following number of cells were analyzed for each condition: mock 23 cells; si-TPM1 12 cells; si-PPP1R12B 15 cells; si-HIPK3 7 cells; si-RAC2 19 cells.

After establishing the importance of TPM1, PPP1R12B, RAC2 and HIPK3 in cell migration and adhesion formation, we next studied the role of these proteins in cellular force application. Previously, we established the quantitative assessment of cellular force application using silicon elastomeric micropillars^22^. Here, we wanted to follow the changes in force application during IGF1-induced MCF7-IGF1R cell migration. In order to facilitate this, MCF7-IGF1R cells were transduced to stably express mCherry-LifeAct, allowing simultaneous visualization of the actin cytoskeleton dynamics together with micropillar bending. Following the siRNA knockdown, MCF7-IGF1R-mCherry-LifeAct cells were seeded on micropillars with an effective Young’s Modulus of 47.2 kPa (bending stiffness of 65.8 nN/μm), stimulated with IGF1 and cellular forces were recorded in time (Fig. 5a). Mock transfected cells rapidly responded to IGF1 stimulation, whereas cells with knockdown of 4 candidates appeared largely unresponsive (Fig. 5b, Supplementary Movies 6,7,8,9,10). The deflection of the individual pillars was used to calculate the force per pillar exerted by the cells. Knockdown of PPP1R12B, RAC2 and HIPK3 caused an increase in force application that remained relatively constant after IGF1 treatment (Fig. 6a,b and Supplementary Fig. S5). For the control and TPM1 knockdown conditions, the average force per pillar initially increased and then remained relatively constant for the duration of the experiment (Fig. 6a). Knockdown of RAC2, PPP1R12B or HIPK3 resulted in a sustained increase in force per pillar (Fig. 6b). To assess whether this reflected a general response of the entire population or whether localized increases in force were involved, we analysed the cumulative distribution function of measured traction forces. In addition to a shift in the population towards higher traction forces following PPP1R12B, RAC2 and HIPK3 knockdown, knockdown of in particular PPP1R12B and RAC2 resulted in wider population distributions (Fig. 6c). This indicated that there is a larger heterogeneity in traction forces, in response to knockdown of these genes.

Lastly, to determine the role of these genes in adhesion force turnover, we determined the autocorrelation of the force magnitudes measured at individual pillars over time. The autocorrelation function provided information on the duration of forces transduced by cellular adhesions, with faster decays indicating that the forces applied through adhesions were changing rapidly. The resulting autocorrelation functions showed the steepest decrease for the mock condition (Fig. 6d,e). Quantification of autocorrelation function halftimes showed fastest force dynamics for mock condition, for which the force halftime was roughly 22 minutes. The halftime was increased by ~1.5 fold after silencing PPP1R12B, HIPK3 or TPM1, although for the latter condition the increase was not significant. Knockdown of RAC2 led to doubling of the force halftime up to >45 minutes (Fig. 6f), which strongly correlated with its most prominent attenuation of focal adhesion dynamics (Fig. 3f).

## Discussion

New insights into cell adhesion and migration are a starting point for identification of drug targets against cancer progression. Our current findings provide a strong correlation between cellular traction force generation and dynamics, cell-matrix adhesion turnover and cell migration. We show that downregulation of the expression of PPP1R12B, HIPK3 and RAC2, in addition to reducing cell migration, results in induction of cellular forces, formation of larger adhesions and alterations in both adhesion and force dynamics. A general set of relations between adhesion dynamics and cell migration have previously been described in a variety of studies, highlighting the connection between adhesion, migration, and force/contractility. While our results confirm a previous reported positive correlation between adhesion size and the magnitude of cellular forces^23^,^24^,^25^, as well as the role of contractility in mediating cell migration^26^,^27^, we now identify a key role for the above-mentioned three novel regulators mediating these processes.

Tumour cells from different origins are diverse in their biology, which is further evidenced based on recent whole genome sequencing. We purposely used a non-motile luminal breast tumour MCF7 cell line to screen for focal adhesion dynamics. Interestingly, and unexpectedly, the hits discovered in this screen showed only a small overlap with an earlier RNAi screen on focal adhesion regulation performed in HeLa cells^28^. Several reasons may explain this difference: (i) the oncogenic programs that define MCF7 and HeLa cells are likely different; (ii) different approaches were used for both the primary and secondary screening; (iii) only 381 of a total of 1269 target genes were shared between the different libraries. Nevertheless, Winograd-Katz *et al*. validated 44 hits, of which 20 were also present in our custom adhesome library, and, more importantly, 13 of those 20 showed significant changes in FA size and/or assembly/disassembly in our study. Using the same siRNA libraries as Winograd-Katz *et al*., a wound healing screen in MCF-10A found and validated 66 genes involved in collective cell migration^29^. Similarly, 28 of those validated genes were also present in our adhesome library, and 20 showed significant changes in FA size in multiple conditions. These comparisons indicate the consistency of the role of these candidate genes in the modulation of FA organization in different cell systems: MCF7 cells (our work) and HeLa cells (Winograd-Katz *et al*.); as well as in different biological read-outs: FA dynamics in MCF7 cells (our work) and MCF-10A cell migration (Simpson *et al*.). Since we have evaluated the effect of knockdown on focal adhesion size in steady state, and during adhesion assembly and disassembly, our data provide new insights in the complex set of molecular determinants that regulate cell-matrix adhesion behaviour.

While we identified key regulators of focal adhesion dynamics, it remains unclear whether our molecules are core components of focal adhesion complexes. Recently a large and integrated proteomic analysis of integrin adhesion complexes from different cell types has revealed a ‘meta-adhesome’ of 2412 proteins and defined a core ‘consensus-adhesome’ of 60 proteins^30^. Knockdown of well-known core components, such as FAK, ILK, TLN1 and ZYX, showed enlarged focal adhesions in our cells (Supplementary Table 2). Although none of our validated hits was present in this core ‘consensus-adhesome’, 8 hits were found in either the ‘meta-adhesome’ or in specific datasets of adhesion assembly and disassembly proteins; these validated hits included ACTR3, ARHGAP24, CAPN6, CD47, CFL1, KEAP1, RAC2 and TPM1. This indicates that these proteins localize to adhesion complexes in context of fibronectin-mediated adhesion. Interestingly, in the proteomic analysis RAC2 was only detected during adhesion assembly, suggesting a role in adhesion maturation. Indeed, our data shows that adhesion assembly is impaired in the absence of RAC2. Both HIPK3 and PPP1R12B were not identified in any of the proteomic analyses, indicating that the enhanced focal adhesion size after siRNA knockdown is most likely a downstream effect of disrupted signalling or contractility.

The IGF1R receptors receives increased attention in the context of breast cancer progression and metastasis formation^21^,^31^,^32^,^33^. We evaluated our candidate genes in MCF7 cells that have increased expression of IGF1R and only increase their motility upon addition of IGF1 (see supplemental movies). 11 candidate genes were rigorously validated for their effect on cell migration. These hits were initially observed under different nocodazole conditions. Importantly, of these 11 hits, 6 showed the same effect in Hs578T, and 7 in the MDA231 (manuscript in preparation); there was an overlap of 3 hits for all cell lines: RAC2, ACTR3 and CD47. This indicates a translation of our findings in MCF7 cells to other more motile cancer cell types. In particular four candidate modulators of focal adhesion dynamics strongly inhibited IGF1R driven MCF7 cell migration which was associated with increased focal adhesion size: PPP1R12B, HIPK3, RAC2 and TPM1. PPP1R12B (larger FAs in control), also known as MYPT2, codes for myosin phosphatase target 2 (MYPT2), which takes part in myosin phosphatase protein complex. The myosin phosphatase protein complex, together with myosin light chain kinase, orchestrates myosin regulatory light chain phosphorylation that in turn controls normal cardiac performance^34^ and is involved in the sarcomeric architecture of actin cytoskeleton^35^,^36^. We show that knockdown of PPP1R12B causes higher forces, induces formation of larger focal adhesions and significantly impairs force turnover. This fits with the inhibitory effect of myosin phosphatase on myosin activity. A closely related isoform, MYPT1, has been shown to play a similar role in actomyosin contractility, adhesion formation and cell migration^37^,^38^, indicating that a homeostatic balance of contractility is required for cell motility. Indeed, our findings suggest that down regulation of PPP1R12B impairs tumour cell migration through its influence on cellular force machinery and adhesion dynamics.

HIPK3 (larger FAs in control) encodes for the protein homeodomain interacting protein kinase 3 and is involved in cell survival and insulin metabolism^39^,^40^. Higher HIPK3 expression correlates with worse prognosis and lower sensitivity to chemotherapy in osteosarcoma and prostate cancer^41^,^42^. HIPK3 has been implicated in JNK signalling^41^,^43^, which is well known for its role in cell migration, adhesion dynamics and cytoskeletal reorganization^44^. Here we show that knockdown of HIPK3 impairs tumour cell migration, induces formation of larger adhesions as well as inducing cellular force application and stability. These findings further indicate HIPK3 as a possible target to impair tumour metastasis as well as inducing higher sensitivity to chemotherapy.

RAC2 (no FA assembly in nocodazole) encodes Ras-related C3 botulinum toxin substrate 2, and is a member of Rho family of GTPases that regulate actin cytoskeleton^45^. Activating mutations of Rac2 have been shown to promote anchorage-independent growth as well as induction of tumour growth^46^. Moreover, Rac2 knock-out macrophages were shown to display altered migration and less podosomal structures, indicative of higher contractility^47^,^48^. Our findings extend these studies: RAC2 is implicated in cancer cell migration and in its absence focal adhesions become static. Furthermore, cellular traction forces at these adhesion sites are increased and very stable.

Even though we could not establish a role for TPM1 (larger FA in control) in traction force dynamics, the TPM1 gene was identified in our primary screen for adhesion dynamics and TPM1 knockdown also attenuated cell migration. The TPM1 gene codes for tropomyosin 1, which takes part in muscle regulation, stabilizes actin cytoskeleton in non-muscle cells and its deregulation is implicated in cardiac illnesses^49^. Opposing findings have been reported for its function in tumour cell migration, which could be explained by different TPM1 isoforms having opposite effects on actin organization^50^,^51^,^52^. Nevertheless, our findings show that down-regulation of TPM1 results in larger adhesions and impaired cell migration suggesting that the main TPM1 isoform targeted in our MCF7 cell lines might be the TPM1λ isoform^52^. Despite the role of tropomyosin in actin organization and previous findings of tropomyosin inducing actomyosin contractility^53^, we did not find significant changes in applied forces or force dynamics upon TPM1 silencing.

In summary, this study aimed to understand the regulation of cell adhesion dynamics and its role in cell migration. We have used microscopy-based assays and analysis to quantitatively assess adhesion size and cell migration speed, and identified 11 candidate genes that regulate these processes. Interestingly, from four of these regulators we tested, three (HIPK3, PPP1R12B and RAC2) were identified to also control the dynamics of traction forces: their downregulation lead to decreased force turnover rates. These results indicate that MCF7 cells have a similar yet highly complex regulatory network that controls force dynamics, cellular adhesion and cell migration, as reported previously for other cell types^26^,^54^. Moreover, it suggests that small dynamic focal adhesions and low dynamic traction forces go hand in hand with an active actin cytoskeleton organization that drives cellular motility. Ultimately, loss of HIPK3, PPP1R12B or RAC2 ensured application of higher cellular traction forces, leading to enlarged focal adhesion and impaired cell migration.

## Materials and Methods

### Cell culture

MCF7 WT (ATCC, Manassas, VA, USA) and IGF1R overexpressing MCF7-IGF1R cells^21^ were cultured in RPMI-1640 medium (Gibco, ThermoFisher Scientific, Breda, The Netherlands) supplemented with 10% foetal bovine serum (FBS, GE Healthcare, Landsmeer, The Netherlands), 25 IU/ml penicillin and 25 μg/ml streptomycin (ThermoFisher Scientific) at 37 °C in a humidified 5% CO_2_ incubator. For visualization of the actin cytoskeleton, cells were transduced using a lentiviral mCherry-LifeAct cDNA expression vector (provided by Dr. Olivier Pertz, University of Basel, Basel, Switzerland), and were cultured in selection medium containing 2 μg/ml puromycin (cat. number 227420500, Acros Organics/Fisher Scientific, Landsmeer, The Netherlands). For live cell imaging, phenol red-free culture medium was used.

### Antibodies and reagents

Mouse anti-paxillin (no. 610052) was purchased from BD Biosciences (Franklin Lakes, NJ, USA). Rabbit anti-phosphorylated (pY118) paxillin (cat. number 44-722G) was from Invitrogen (ThermoFisher Scientific). Mouse anti-vinculin (V-9131) and mouse anti-tubulin (T-9026) were obtained from Sigma-Aldrich (St. Louis, MO, USA). Mouse anti-GAPDH (sc-32233) was purchased from Santa Cruz (Dallas, TX, USA). Rhodamin phalloidin and goat anti-mouse Alexa-488 conjugated secondary antibodies were from Molecular Probes (Invitrogen/ThermoFisher Scientific). Goat anti-rabbit CY-3 and goat anti-mouse Alexa647 conjugated secondary antibodies were from Jackson ImmunoResearch (West Grove, PA, USA). Nocodazole was purchased from Fluka (Zwijndrecht, The Netherlands).

### siRNA reagents

All siRNA reagents and siGENOME siRNAs were obtained from Dharmacon (Lafayette, CO, USA). A custom designed SMARTpool siRNA library targeting 569 genes with known or predicted roles in cell adhesion was used (Supplementary Table 1). SMARTpools consisted of four different siRNA sequences targeting the same gene. DharmaFECT 4 was used as transfection reagent.

### RNAi screen protocol

Glass bottom 96-well plates (Greiner Bio-One, Frickenhausen, Germany) were coated with 10 μg/ml Collagen type 1 (isolated from rat tails) for 1 h at 37 °C, after which plates were washed twice with PBS and used. A 50 nM reverse transfection was performed according to manufacturer’s guidelines. Complex time was 20 min and 10,000 MCF7 WT cells were added. Transfection was performed in duplicate. Each plate contained negative controls (no siRNA, mock, siGFP (D-001300-01, Dharmacon) and non-targeting Control #2 (D-001210-02, Dharmacon)), a positive control (si-DNM2) and transfection controls (si-KIF11, si-PXN and si-GLO Green). Plates were placed in the incubator and the medium was refreshed after 20 h.

32 h after transfection cells were placed on overnight serum starvation. The next day, a nocodazole assay was performed, in which cells were exposed to one of three conditions: 1) 0.025% DMSO in starvation medium for 6 h, 2) 10 μM nocodazole in starvation medium for 4 h, or 3) 4h 10 μM nocodazole followed by a 2 h washout with 0.025% DMSO in starvation medium. Transfection controls (si-KIF11, si-PXN and si-GLO Green) were not exposed. After treatment, cells were fixed in 4% buffered formaldehyde for 10 min, and washed thrice with PBS.

Fixed cells were blocked in TBP (0.1% Triton X-100 (Sigma-Aldrich), 0.5% BSA (Sigma-Aldrich) in PBS) for 1 h at RT. Immunostaining was performed for phosphorylated (pTyr-118) paxillin and vinculin, followed by secondary antibodies conjugated with CY-3 or Alexa488, respectively. Hoechst 33258 (Sigma-Aldrich) was used to visualize nuclei.

### Automated microscopy

Microscopy was performed on a Nikon Eclipse T*i* confocal microscope (Nikon, Amsterdam, The Netherlands) and this system included a 37 °C incubation chamber, an automated xy-stage, an integrated Perfect Focus System (PFS) and 408, 488 and 561 Argon lasers. The system was controlled by Nikon’s EZ-C1 software (version 3.90). All images were acquired using a Plan-Apochromat 20x objective with 0.75 NA, at a resolution of 512 × 512 pixels, with a pixel dwell time of 7 μs and 4x scanner zoom, unless stated otherwise. The final image is an average of two scans for both 488 and 561 signals and a single scan for 408.

For automated imaging, a custom-written macro was used within EZ-C1. This macro was able to search for cells, then focus on the focal adhesions and acquire an image, for any given number per well. The three corner wells of a 96-well plate were selected and the coordinates (x, y, z and PFS-value) were saved. The macro then generated at random coordinates for all positions where the image would be acquired. Using the Perfect Focus System, the software searches randomly for cells in Hoechst channel (408-laser) until a certain threshold is met, i.e. a number of cells per well (pre-set). The PFS is then turned off, and using a custom autofocus it focuses on the focal adhesions. Once the optimal focus is found, the system acquires the image and then continues with the next position. Between 5 and 8 images per well were acquired.

### Image analysis

Image analysis was implemented using ImageJ version 1.43 h (http://imagej.nih.gov/ij/). Acquired images were split into the original channels and the nuclei channel was used to remove empty images. The analysis was performed for one channel at a time. First, the image is passed through a Gaussian filter to normalize the CCD signal and a rolling ball is applied to remove noise. Next, segmentation was performed based on a watershed masked clustering algorithm^55^,^56^. Objects smaller than 4 pixels are ignored. Labelled objects are converted to numerical data, for several FA features: area, perimeter, extension, dispersion, elongation, orientation, compact factor and average intensity. The entire segmentation is run twice; once for the green channel (vinculin) and once for the red channel (p-Tyr118-paxillin).

### Data analysis

Focal adhesion data was analysed using Matlab (Mathworks, Natick, MA, USA). Date from duplicate wells were combined and measurements of each individual focal adhesion were used for statistical analysis. A two-sample Kolmogorov-Smirnov (KS) test was used to compare distributions of FA sizes. The KS-test returned a score of −1, 0 or +1, indicating a decrease, no change, or increase in FA size, respectively. A D-statistic and p-value were calculated as measurement of the shift between distributions and its significance. For simplicity, hit selection was based on the analysis of vinculin-positive adhesions. Thresholds for hit selection were empirically defined for each condition and described in the results; this was followed by visual inspection of images for hit deselection.

### Live cell migration

MCF7-IGF1R cells^21^ were used for live cell migration assays. Transfections were performed as described above, with 15,000 cells in a standard 96-well culture plate. After 56 h, the transfected MCF7-IGF1R cells were replated onto collagen-coated glass bottom plates and were allowed to adhere overnight. Cells were switched to starvation medium and pre-exposed for 45 min to 100 ng/ml Hoechst 33342 (Fisher Scientific). After refreshing the medium, cells were placed on a Nikon Eclipse T*i* microscope fitted with a 37 °C incubation chamber, 20x objective (0.75 NA, 1.00 WD), automated stage and PFS system. Three positions per well were manually selected, and Differential Interference Contract (DIC) and Hoechst images were captured with a DS-Qi1MC CCD camera with 2 × 2 binning (pixel size: 0.64 μm). Images were acquired every 10 to 14 minutes for 3 h, after which cells were stimulated with 100 ng/ml IGF-1 (Increlex, Ipsen, Basking Ridge, NJ, USA) and image acquisition continued for 7 h using NIS software (Nikon). All images were sorted using custom-made R-scripts. Image analysis was performed using CellProfiler^57^ (Broad Institute). Briefly, images were segmented using a watershed masked clustering algorithm^56^, after which cells were tracked based on overlap between frames. Tracking data was organized and analysed using in-house developed R-scripts (package H5CellProfiler, Wink *et al*., manuscript in preparation^58^) to obtain single cell migration data. For each cell, the position shift between frames was used to calculate the average speed per cell. Average single cell migration speeds were plotted using GraphPad Prism 6.0 (GraphPad Software, San Diego, CA) and changes in migration speed were evaluated by comparing cell populations (Kruskal–Wallis with Dunn’s multiple comparisons post-test). For all live microscopy, experiments were performed in duplicate and results were considered significant if p-value < 0.05 for all experiments.

### Traction force microscopy with silicon elastomeric micropillar post arrays

Micropillars were used for cellular traction force measurements according to methodology described previously^23^. MCF7-IGF1R-mCherry-LifeAct cells were transfected as described above. After 65 h, cells were used for micropillar experiments. Nanolithography with PDMS was performed to create pillars of 4.1 μm height, 2 μm diameter, 4 μm centre-to-centre distance in a hexagonal lattice with spacers on the side. Pillars were calculated to have a bending stiffness of 65.8 nN/μm and an effective Young’s modulus of 47.2 kPa^23^. ECM stamping was performed using a flat piece of PDMS pre-incubated with a 40 μl mix of [50 μg/mL unlabelled fibronectin (Sigma Aldrich) and 10 μg/mL Alexa647-conjugated fibronectin (Invitrogen)]. Following blocking with 0.2% Pluronic (F-127, Sigma Aldrich) in PBS for 1 hour, transfected cells were pipetted on the pillar array in warm medium and were incubated for 2 hours. Cells were subsequently serum starved for 3 hours. For imaging the pillars with cells on top, the pillar arrays were placed upside down in a 24 well glass bottom plate (Greiner Bio-One), mounted on a Nikon Eclipse T*i* microscope, stimulated with 100 ng/mL IGF1 and imaged every 5 minutes for 400 minutes in scanning confocal mode together with a 20x magnification 0.75 NA dry air lens with internal 1.5x magnification and 4.184 scanner zoom to obtain a pixel size of 0.2 μm.

Forces were calculated with approximately 2 nN precision from the pillar channel using specifically designed Matlab scripts as described previously^23^. Briefly, deflections of individual pillars were calculated by relating the exact pillar locations determined from the labelled fibronectin fluorescence image to the reference undeflected hexagonal grid. Movies were generated and manually checked for movies with deflections that had high signal-to-noise ratio and to remove cells that died or divided. Cell masks were generated from the mCherry-LifeAct channel by first passing the image through a Gaussian low pass filter, subtracting the background intensity and running the image through a sobel and a log-edge detection algorithm followed by image dilation and hole filling each time. Pillars were followed through the movie with in-house written Matlab script that matched pillars in subsequent frames (or 2 frames apart if a match was not found in first iteration) that were closer than 2 μm. This enabled tracking more than 90% of the pillars for the duration of the movie. Pillars coupled to cells that showed the top 5% deflection for the duration of the imaging were taken for further analysis. Average force per pillar was determined by averaging the pillar deflections for the whole duration of the movie for all selected cells. Autocorrelation was calculated for top 5% deflected pillars per movie using Matlab acorr function, averaged per condition and an exponential function was fit for the first 2 hours (25 data points) to obtain the half time.

### Western Blot

MCF7 WT and MCF7-IGF1R cells were transiently transfected as described above. After 72 h, cells were lysed using SDS protein buffer (125 mM Tris/HCl pH 6.8, 20% glycerol, 4% SDS and 0.2% bromophenol blue). Proteins were resolved by SDS-PAGE and transferred to polyvinylidine difluoride membranes. Membranes were blocked in 5% BSA in Tris-buffered saline with 0.05% Tween-20 (TBS-T), followed by overnight incubation with primary antibodies, washing, and 1 h incubation with Alexa647-conjugated secondary antibodies. Fluorescence was detected with a LAS 4000 imager (GE Healthcare).

### Statistical Analysis

Normality of migration measurements was tested using Kolmogorov–Smirnov’s test, d’Agostino and Pearson’s test and Shapiro–Wilk’s test using GraphPad Prism 6.0. A data set was considered normal if found as normal by all three tests. Significance was calculated according to the method indicated at individual figure legends using GraphPad Prism 6.0 or Matlab.

## Additional Information

**How to cite this article**: Fokkelman, M. *et al*. Cellular adhesome screen identifies critical modulators of focal adhesion dynamics, cellular traction forces and cell migration behaviour. *Sci. Rep.* **6**, 31707; doi: 10.1038/srep31707 (2016).

## Supplementary Material

Supplementary Information

Supplementary Movie 1

Supplementary Movie 2

Supplementary Movie 3

Supplementary Movie 4

Supplementary Movie 5

Supplementary Movie 6

Supplementary Movie 7

Supplementary Movie 8

Supplementary Movie 9

Supplementary Movie 10

Supplementary Table 1

Supplementary Table 2

Supplementary Table 3
