# Supplementary material for: Cellular adhesome screen identifies critical modulators of focal adhesion dynamics, cellular traction forces and cell migration behaviour
Source: Sci Rep. 2016 Aug 17;6:31707. doi: 10.1038/srep31707 (PMC4987721; doi:10.1038/srep31707)
Supplement: Supplementary Information [file srep31707-s1.pdf]

Cellular adhesome screen identifies critical modulators of focal adhesion dynamics, cellular traction forces and cell migration behaviour

SUPPLEMENTARY INFORMATION

Michiel Fokkelman[1], Hayri E. Balcıoğlu[1], Janna E. Klip[1], Kuan Yan[2], Fons J. Verbeek[2], Erik H.J. Danen[1], Bob van de Water[1,*]

[1] Leiden Academic Centre for Drug Research, Division of Toxicology, Leiden University, The Netherlands.

[2] Imaging & Bioinformatics, Leiden Institute of Advanced Computer Science, Leiden University, The Netherlands.

* Author for correspondence (b.water@lacdr.leidenuniv.nl)

Figure S1

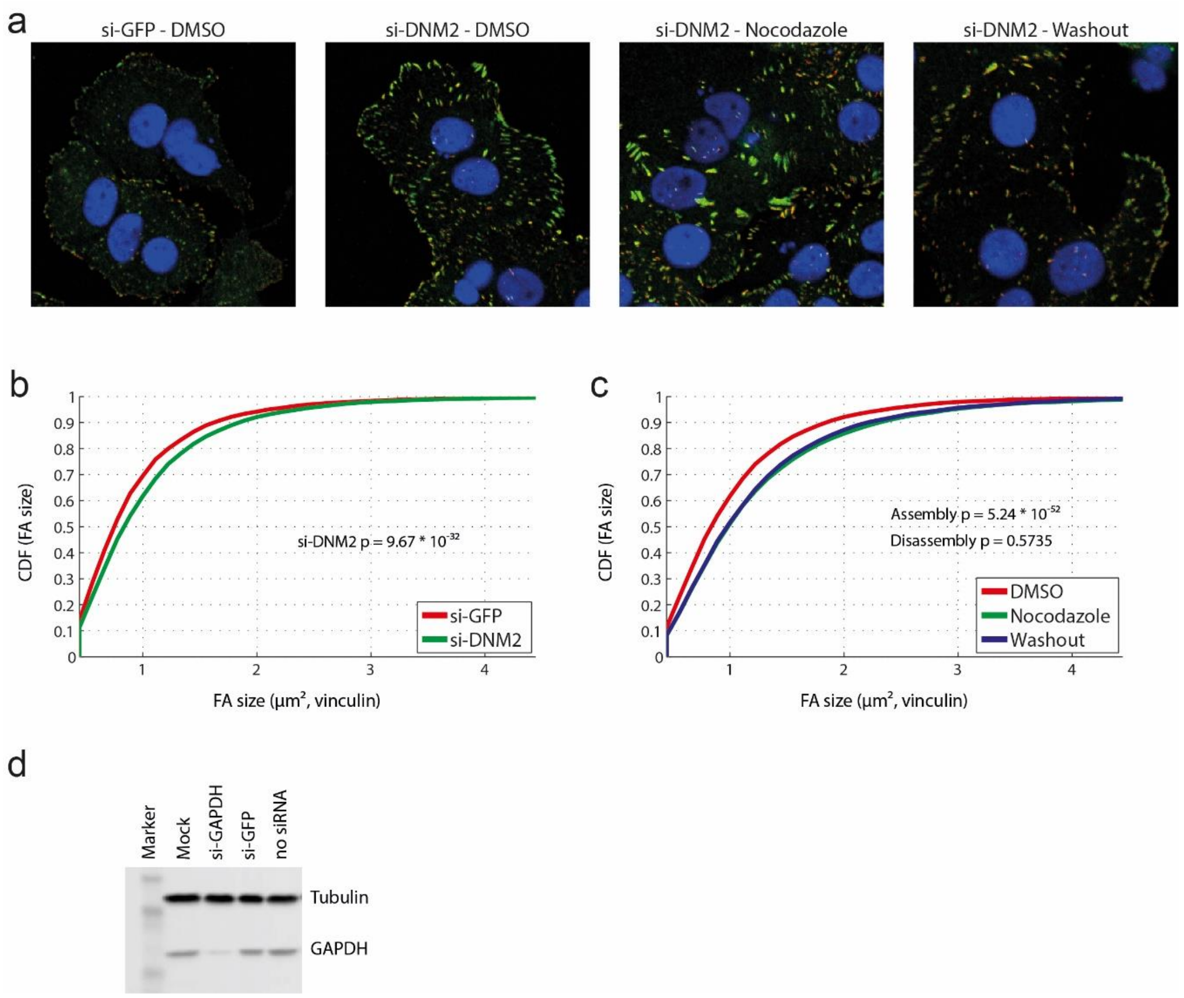

Supplemental Figure 1: Knockdown of DNM2 results in enlarged focal adhesions and impaired focal adhesion disassembly. (**a**) Representative images of focal adhesions in MCF7 cells transfected with si-GFP or si-DNM2. Cells were treated as indicated and adhesions were visualized by staining for vinculin and pTyr118-paxillin. (**b**) Focal adhesion size distribution of si-DNM2 knockdown in DMSO condition was compared to siGFP control cells using a two-tailed KS-test. Knockdown of DNM2 results in enlarged adhesions. (**c**) Comparison of adhesion size in DNM2 depleted cells show typical nocodazole-induced adhesion assembly followed by impaired adhesion disassembly. (**d**) Knockdown efficiency was confirmed by Western Blot analysis.

Figure S2

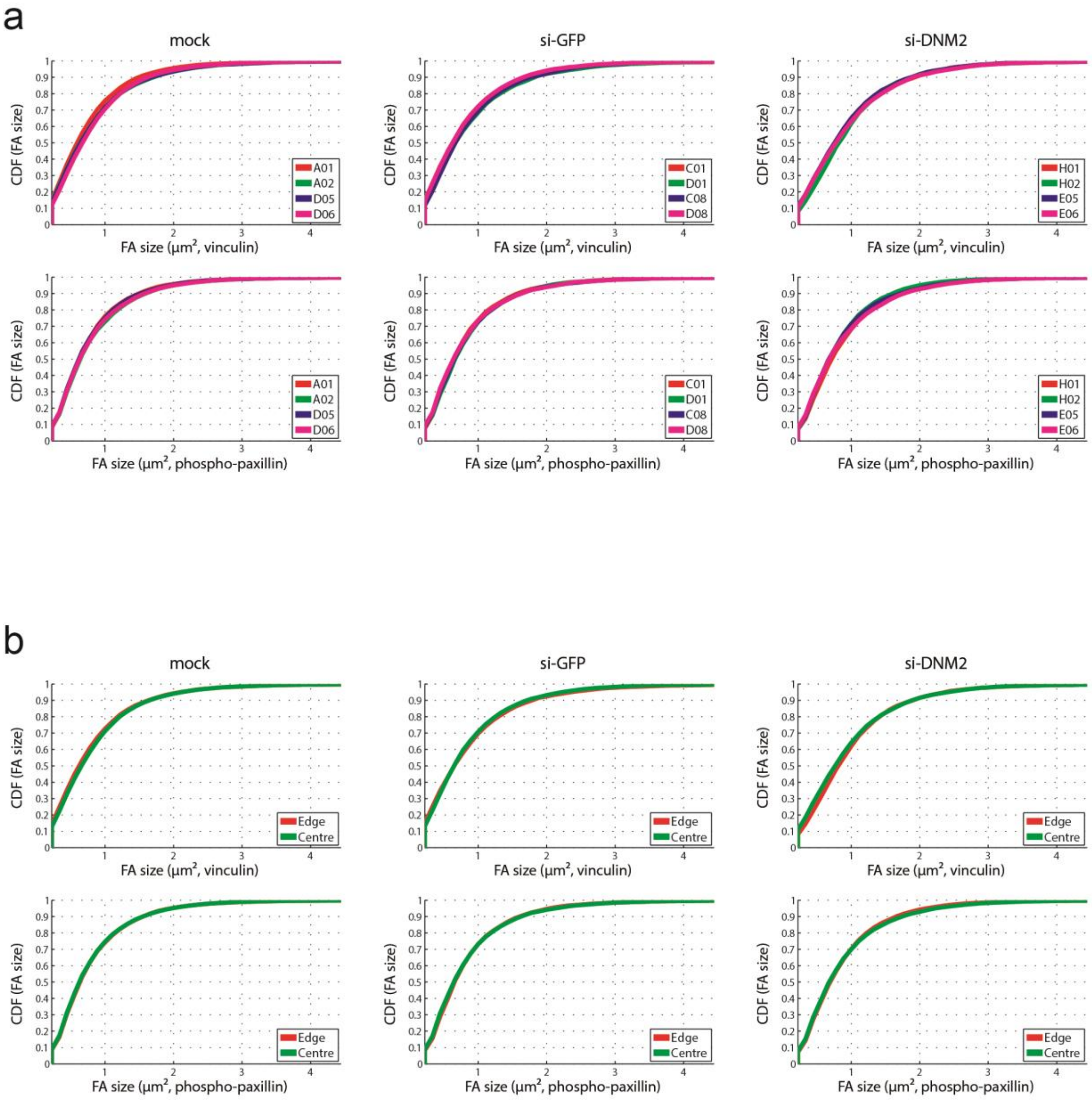

Supplemental Figure 2: Focal adhesion size distributions from different wells show little variation and no positional or edge effects. (**a**) Focal adhesion size distributions of mock, si-GFP and si-DNM2 transfected cells from different wells. Top graphs show vinculin-stained adhesions, bottom graphs shows phospo-paxillin. (**b**) Focal adhesion size distributions of mock, si-GFP and si-DNM2 transfected cells from duplicate wells at the edge or center of the plate. Top graphs show vinculin-stained adhesions, bottom graphs shows phospo-paxillin.

Figure S3

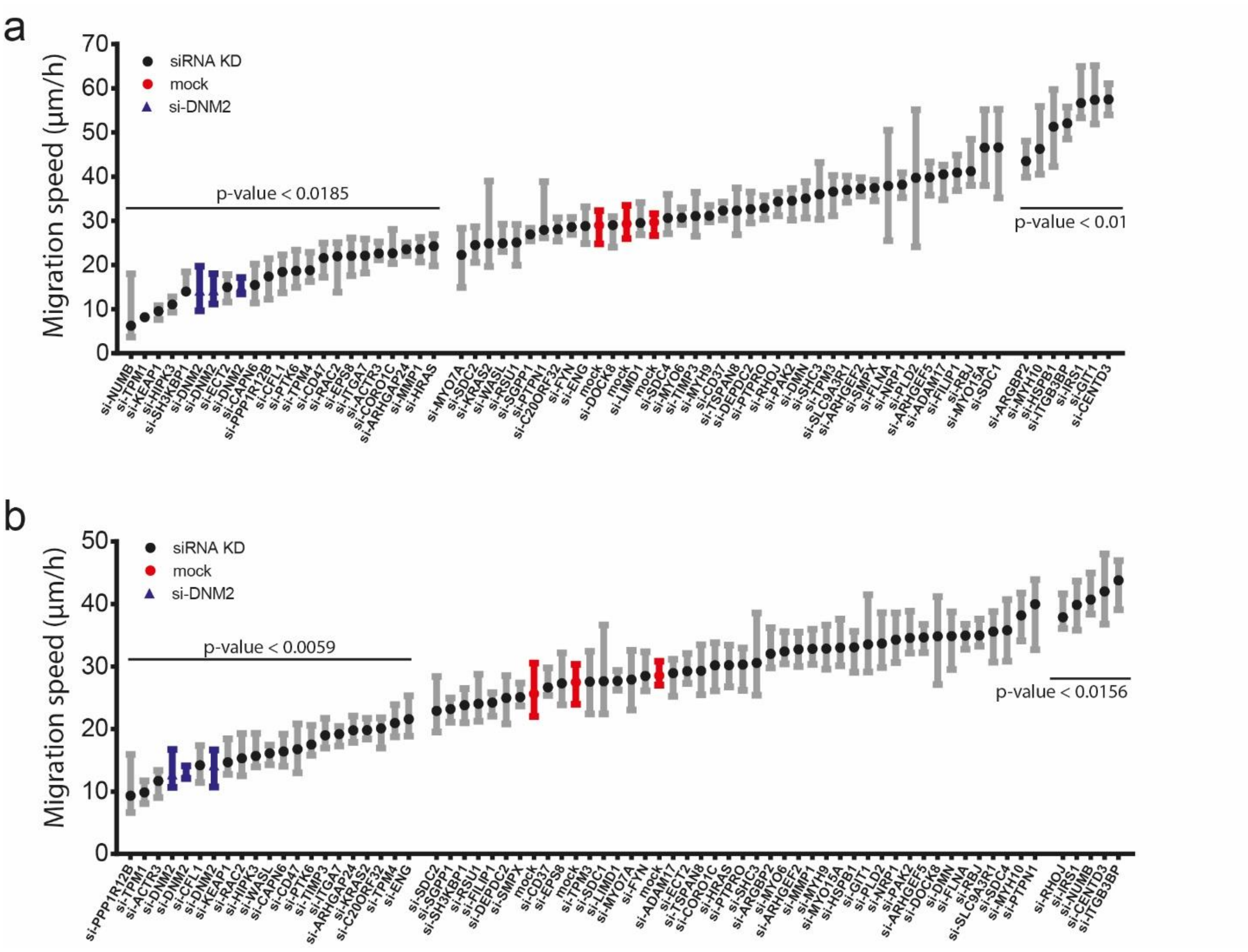

Supplemental Figure 3: Quantification of single cell migration speed of MCF7-IGF1R cells after knockdown of 64 hits. (**a,b**) MCF7-IGF1R cells were transfected with siRNAs and cell migration was assessed by live microscopy. Two independent experiments are shown in **a** and **b**. Only siRNAs that showed consistent and significant results in both experiments were further validated by deconvolution experiments (Supplemental Fig. S3). Median ± 95% confidence interval is shown and cell populations were compared by Kruskal-Wallis test with Dunn's post correction test. P-values below 0.05 were considered significant.

Figure S4

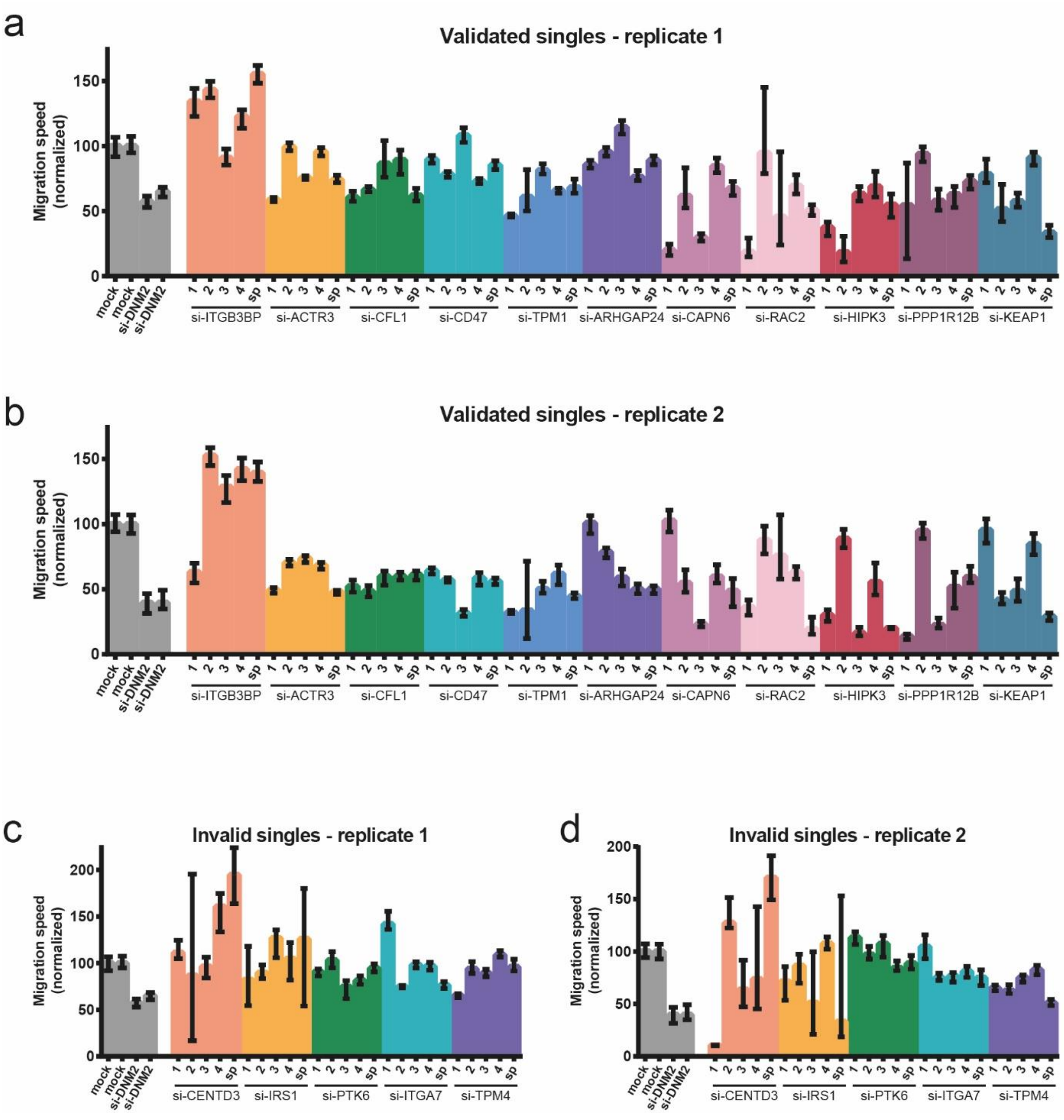

Supplemental Figure 4: Quantification of single cell migration speed of MCF7-IGF1R cells after knockdown of 16 hits using single siRNA sequences and smartpools. MCF7-IGF1R cells were transfected with siRNAs and cell migration was assessed by live microscopy. Independent experiments are shown in **a**, **b**, **c** and **d**. Hits with at least 2 single siRNAs that showed consistent and significant results in both experiments were considered validated. Normalized median ± 95% confidence interval is shown and cell populations were compared by Kruskal-Wallis test with Dunn's post correction test. P-values below 0.05 were considered significant.

Figure S5

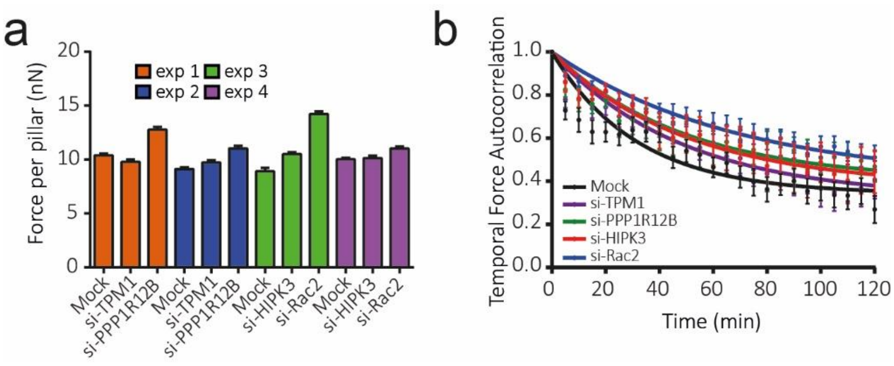

Supplemental Figure 5: Knockdown of PPP1R12B, HIPK3 and RAC2 increases cellular force application and reduces force dynamics. (**a**) Bar graphs showing mean and 95% confidence interval of force per pillar for indicated SMARTpools in independent experiments. (**b**) Force per pillar autocorrelations and corresponding fits using single exponential decay function for indicated knockdowns. Half-times were calculated from the exponential fits and are shown in Figure 6f.

Movies

Movies 1 – 5: live cell migration of IGF1-stimulated MCF7-IGF1R cells. Movies 1 to 5 show mock control, knockdown of HIPK3, PPP1R12B, RAC2 and TPM1, respectively.

Movies 6 – 10: traction force microscopy with MCF7-IGF1R cells on micropillar arrays. Movies 6 to 10 show mock control, knockdown of HIPK3, PPP1R12B, RAC2 and TPM1, respectively.

Movies are deposited online on Figshare (https://figshare.com/s/5bd0c2c1bb35285dc304).

Table 1

Complete table of siRNAs in Adhesome library

Table 2

Screen results

Table 3

List of primary hits
